# Q&A: Cellular near death experiences—what is anastasis?

**DOI:** 10.1186/s12915-017-0441-z

**Published:** 2017-10-24

**Authors:** Gongping Sun, Denise J. Montell

**Affiliations:** Molecular, Cellular, and Developmental Biology Department, University of California, Santa Barbara, CA USA

## Abstract

Apoptosis is a form of programmed cell death that is carried out by proteolytic enzymes called caspases. Executioner caspase activity causes cells to shrink, bleb, and disintegrate into apoptotic bodies and has been considered a point of no return for apoptotic cells. However, relatively recent work has shown that cells can survive transient apoptotic stimuli, even after executioner caspase activation. This process is called anastasis. In this Q&A, we answer common questions that arise regarding anastasis, including how it is defined, the origin of the name, the potential physiological consequences, molecular mechanisms, and open questions for this new field of study.

## What is anastasis?

**Fig. 1.**
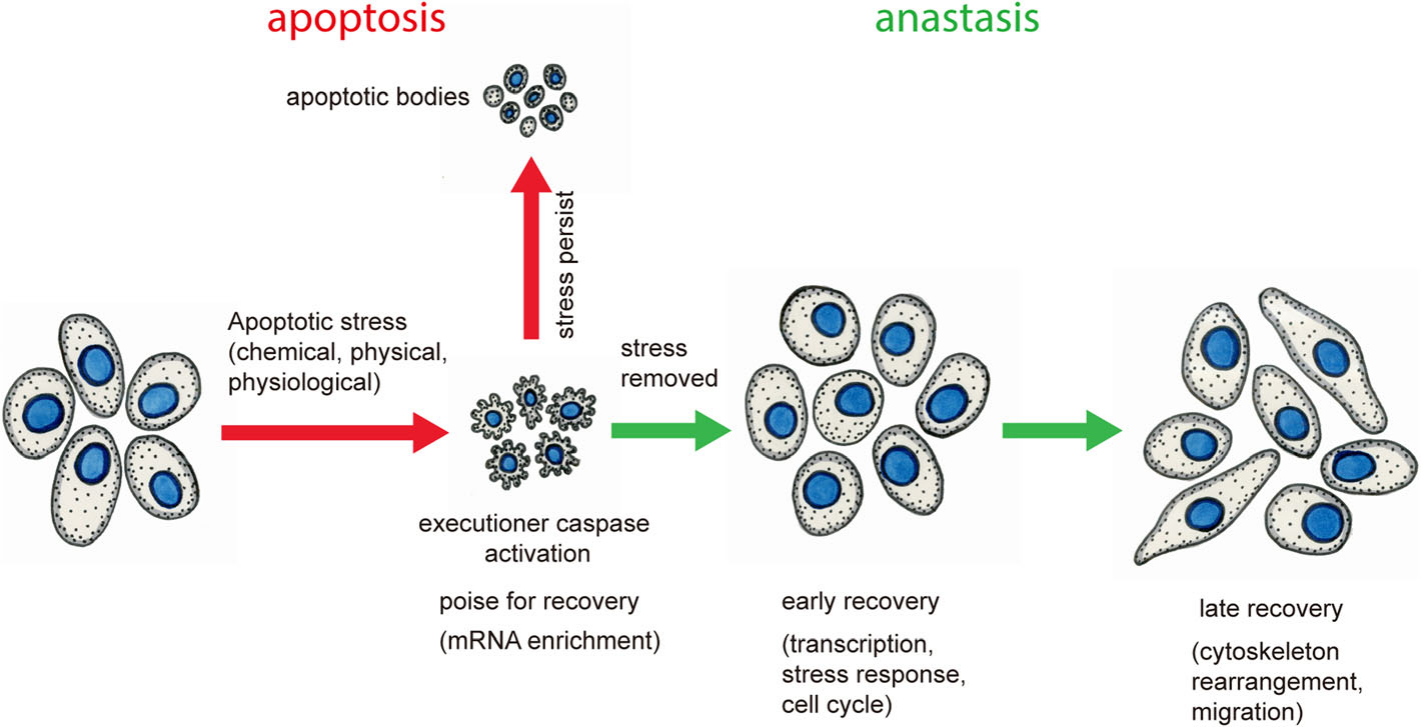
Apoptosis and anastasis. When cells experience a potentially lethal dose of a chemical stress, they simultaneously activate a pro-survival stress response and initiate the apoptotic process, activating caspase 3. They poise for recovery by enriching some mRNAs encoding survival proteins. If the stress persists, the apoptotic process dominates and the cells dissociate into apoptotic bodies. If the stress is relieved, cells undergo a two-stage recovery. The early recovery involves transcription initiation, stress response, and re-entry into the cell cycle. The late recovery involves cytoskeleton rearrangement and cell migration. Adapted from [9], ©2017 Sun et al. The Journal of Cell Biology. 216:3355–3368; DOI:10.1083/jcb.201706134

Anastasis refers to cellular recovery from the brink of apoptotic death. Anastasis is a process by which cells survive executioner caspase activation following transient exposure to a lethal dose of an apoptotic stimulus. It was first named in 2012 [1]. In this study, we applied chemical toxins to mammalian cells in order to induce apoptosis in a variety of cell types and waited until they showed classic apoptotic hallmarks including activation of caspase 3, cell shrinkage, and membrane blebbing. If cells were left in the toxins, the vast majority of them died. However, removing the chemical stress after a few hours by replacing the growth medium allowed most cells to recover a relatively normal morphology (Fig. 1). This recovery is called anastasis, which is a Greek word meaning ‘rising to life’. The word apoptosis is also derived from Greek roots and means falling to death, like leaves falling from trees or petals from flowers [2].

## Is anastasis really a new discovery?

The discovery of anastasis shows that the activation of the executioner caspases is not ‘the point of no return’ in apoptosis, which is a new concept. Apoptosis is a cell suicide process that was initially described as a series of morphological changes resulting in cell fragmentation into apoptotic bodies and their subsequent removal by phagocytosis [2]. After decades of study, the core molecular mechanisms regulating apoptosis are well-established. While many different stimuli can initiate apoptosis, they all ultimately cause the activation of executioner caspase enzymes [3]. The activation of the executioner caspases during apoptosis occurs rapidly. In HeLa cells treated with apoptosis inducers, such as staurosporine, or TRAIL together with cycloheximide, executioner caspase activity reaches its maximal level within 20 min after the onset of activation [4–6]. Activation of the executioner caspases results in irreversible proteolysis of numerous targets, which leads to dismantling of the cell [7]. Thus, the classic view of apoptosis has been that, after the activation of the executioner caspases, death is inevitable.

Nevertheless, retrospectively, hints of anastasis can be found in the literature. For example, a study in *Caenorhabditis elegans* showed that some cells that are normally destined to die by apoptosis during development can live and go on to differentiate into neurons when their removal by phagocytosis is prevented in animals bearing mutations in engulfment genes [8]. The cells presumably recover from caspase activation, though this has not been demonstrated directly. The fraction of cells that recover increases from 3% to > 50% when one copy of the gene encoding executioner caspase is mutated in the same animals with defective phagocytosis, which shows that this process is sensitive to caspase levels, thus hinting that a threshold level of executioner caspase expression is required to complete apoptosis.

## What kinds of apoptotic stimuli permit anastasis?

Recovery from caspase activation after exposure to multiple types of stimuli has been observed. These include: 1) chemical apoptosis inducers such as ethanol, DMSO, staurosporine, jasplakinolide, and cucurbitacin [1, 9]; 2) the death-inducing ligand TNFα combined with cycloheximide [9]; 3) physical inducers such as cold shock [10]; 4) physiological stress such as protein starvation [10]. Further study will be needed to uncover the diversity of apoptotic inducers from which cells can recover.

## What kinds of cells can undergo anastasis?

In vitro, anastasis has been observed in multiple cultured cancer cell lines, including the cervical cancer cell line HeLa and the glioma cell line H4. Anastasis has also been observed in immortalized non-cancer cell lines like NIH3T3 and in primary cells isolated from liver and heart [1, 9, 11]. In vivo, evidence exists for anastasis in epithelial tissues in *Drosophila*, mammalian cardiac myocytes and neurons, and neuronal cells of *C. elegans*. Despite the fact that anastasis has only relatively recently been discovered, the variety of cell types and animals in which it has been observed suggests that it may be general to many if not all types of cells.

## When and where does anastasis occur in vivo?

To determine where and when cells survive caspase activation in vivo, we developed a genetically encoded sensor in *Drosophila* that converts transient executioner caspase activation to permanent expression of a fluorescent protein. Therefore, all cells that survive executioner caspase activation as well as their progeny are labeled. Using this tool, we found that many cells survive caspase activation during larval and pupal development as well as in the adult, in the absence of any external stress [12]. In contrast, few if any cells of the embryo survive caspase activation, suggesting that the ability to undergo anastasis changes during development.

In retrospect, several published examples may also meet the definition of in vivo anastasis including, for example, cardiac myocytes exposed to transient ischemia in mice and rabbits [13]. Mouse neurons over-expressing Tau protein survive caspase-3 activation and develop tangles, though it is not clear if apoptosis was initiated [14]. The discovery of anastasis in mammals and flies suggests that it is an ancient and evolutionarily conserved cellular process.

## What is developmental anastasis?

In *Drosophila*, we were surprised to find cells that survive caspase-3 activation in larval imaginal discs and the optic lobes of the larval brain, where the executioner caspases are dispensable for tissue development. In such tissues, where cell survival of caspase activation appeared sporadically in both space and time, we propose that the cells have undergone ‘developmental anastasis’ [12]. Since it is mainly observed in proliferating tissues, we hypothesize that, in rapidly growing tissues, some cells may randomly experience transient stresses, such as a shortage of growth factors or mechanical compression, leading to caspase activation. Some cells with active caspase die, whereas others ultimately survive transient caspase activation through anastasis. Consistent with this interpretation, cell crowding causes caspase activation during development of the pupal notum in *Drosophila*. Many of the caspase-positive cells are extruded basally and die by apoptosis, whereas others exhibit transient caspase activation and yet survive [15]. This seems to be a particularly clear example of what may be a general phenomenon. That is, during development, cells compete with one another for resources including space, nutrients, and growth factors. Cells lacking sufficient resources initiate the apoptotic pathway. However, the death of some cells may actually relieve the stress on the others, liberating resources and allowing them to recover.

## What is the difference between anastasis and non-apoptotic caspase activation?

It has been clear for some time that some cells activate either initiator or executioner caspases as part of normal development, without causing cell death (recently reviewed in [16]). In some cases, the caspase activities are localized to particular compartments in the cell and result in partial cellular destruction. An example of this type of non-apoptotic caspase function occurs in sperm maturation, dendrite remodeling, and nuclear destruction in mature lens fiber epithelial cells and red blood cells. What these examples have in common with anastasis is sublethal activation of caspases and survival of the cell. What is different is that the cells are not at risk of dying. In anastasis, cells experience a stress capable of killing them if it persists long enough, whereas in cells in which caspase activity performs a normal function, the activity level and/or localization are restricted so as to prevent death. The mechanisms that restrict caspase activity are not yet fully understood.

Both cells that survive non-apoptotic caspase activation and cells that undergo anastasis activate CasExpress, the genetically encoded reporter of cells that survive caspase activation [12]. Operationally we can distinguish cells with non-apoptotic CasExpress from cells that have undergone anastasis. A common characteristic of non-apoptotic caspase activation is that it occurs in a specific stage of development and in every cell of the same type in the tissue. In contrast, developmental anastasis occurs in a random fraction of cells, sporadically over time, in tissues with sporadic apoptosis [12].

## Is anastasis simply arrest of the apoptotic process?

No, transcriptome profiling of ethanol-exposed HeLa cells showed that anastasis is an active process composed of two distinguishable stages, rather than simply arrest of the apoptotic process. The early stage involves transcription of many transcription factors, stress response, re-entry into the cell cycle, and proliferation. Cells in the late stage undergo cytoskeletal rearrangement and morphological change and become more migratory [9]. So, the survivors are not ‘zombies’ but can proliferate and migrate.

## Are there other examples of cells surviving caspase 3 activity under stress and are these the same as or different from anastasis?

In 2015, Liu et al. reported that MCF10A cells can survive with activated caspase 3 after exposure to low-dose ^56^Fe ion irradiation (<3 Gys) [17]. Recently, the same group reported some MCF10A cells can escape cell death induced by Myc overexpression even though caspase 3 was activated [18]. Ichim et al. found ABT-737, a prototypic BH3-mimetic compound, induced mitochondrial outer membrane permeabilization (MOMP) in a small fraction of mitochondria, leading to sublethal caspase 3 activation [19]. In these studies, although caspase 3 was activated, no other apoptotic hallmarks were reported, so it is unclear whether the apoptotic process was initiated. In contrast, in experiments designed to detect anastasis, we observe cell shrinkage, membrane blebbing, and Annexin V staining in the majority of cells treated with the apoptotic inducer, prior to washing and recovery [1, 9]. Although the majority of work in the apoptosis field suggested that caspase activation was an all-or-nothing event [5–7], other work implies that different cells have different thresholds for the lethal level of caspase 3 activity [20].

## Is anastasis the only example of a reversible cell death process?

No. Cells can recover from a variety of near-death experiences. A process called resuscitation was recently discovered, which refers to the recovery from near death in cells undergoing necroptosis [21]. Even the dramatic form of cell death known as entosis, in which one cell swallows another alive, turns out to be reversible [22]: the internalized cell can emerge to live again.

## What are the molecular mechanisms that allow cells to recover from the brink of death?

Whole transcriptome sequencing of HeLa cells during recovery from ethanol-induced caspase activation revealed that anastasis is a complex process involving burst of transcription, stress responses, survival, proliferation, and production of angiogenic factors. More than 1000 genes are upregulated during early recovery [9]. Interestingly the molecular features of anastasis overlap to some extent, but are also clearly distinguishable from, the molecular response of cells recovering from a different type of growth-arresting stress, autophagy [9]. Pharmacological inhibition of transcription, BCL-2, XIAP, MDM2, or heat shock protein 90 suppresses recovery from ethanol-induced caspase activation in primary cells, suggesting the requirement for new transcription and elevated survival and stress response proteins in anastasis [1]. Further study is needed to identify the key regulators of anastasis.

The transcriptional profiles of cells undergoing anastasis revealed that the transcripts of some genes that were highly upregulated during early recovery accumulated already before removal of the apoptotic stimulus. This finding suggests that cells poise for recovery while under apoptotic stress, by enriching the mRNAs for specific genes, which may facilitate rapid recovery after removal of the stress (Fig. 1). One example is Snail. The mRNA encoding Snail is enriched in apoptotic cells and further elevated during early recovery. Cells with Snail knocked down show significantly lower capacity to recover from ethanol or staurosporine-induced caspase activation than control cells. However, inhibition of TGFβ signaling, which blocked Snail expression after 4-h recovery, did not affect survival, confirming that it is the ‘poised’ Snail that is essential [9].

## Does anastasis cause any permanent changes in cells?

In our previous study, we found a small fraction of NIH 3T3 fibroblast cells that survive caspase activation after transient exposure to a lethal dose of ethanol showed chromosomal aberrations and underwent oncogenic transformation [1]. However, it is not known if the majority of cells that have undergone anastasis retain any genetic or epigenetic mark of their near-death experience.

## Why might cells have evolved the ability to undergo anastasis?

The discovery of anastasis unveils a new survival strategy in both normal and cancer cells. Apoptosis appears to have evolved as a mechanism to eliminate cells that are potentially harmful such as autoreactive T cells or cells with DNA damage, so what is the benefit of rescuing cells from the brink of apoptotic cell death when such rescue runs the risk of preserving cells that might do harm? A working hypothesis is that anastasis evolved to limit the permanent tissue damage that would otherwise occur in response to a severe but transient injury, radiation exposure, or chemical stress.

## What are the potential implications of anastasis for cancer?

Radiation and many commonly used chemotherapy drugs induce apoptosis (reviewed in [23, 24]) and are delivered transiently due to their toxicity. So, if tumor cells coopt the normally beneficial recovery process of anastasis, and thus escape therapy-induced cell death, anastasis could underlie relapse following treatment. Anastasis has been observed in cancer cell lines [1, 9], and a fraction of cervical cancer cells that have undergone anastasis exhibit changes in cell morphology and become more migratory [9]. Therefore, by promoting survival and migration, anastasis in tumor cells may contribute to metastasis. Thus, studying the mechanisms underlying anastasis and finding methods to enhance or inhibit it may provide new treatment strategies for cancer.

## What are the future directions for anastasis research?

There are many open questions for anastasis research. What are the earliest events in the process that allow cells to recover even before new gene transcription takes place? Are there permanent epigenetic changes in cells that have undergone anastasis? Are cells that have undergone anastasis more—or less—resilient to future stresses? What is the true point-of-no-return during apoptosis? Can we identify small molecule or biological enhancers or inhibitors of anastasis for use in treating heart attacks, strokes, cancer, and neurodegeneration? Are there cell-type-specific and/or drug-specific features of anastasis that we can exploit therapeutically? Is there a core, conserved mechanism that is the same in all cell types? These and many other questions will be the subject of ongoing and future research in this exciting new field of study.

## References

[1] Tang HL, Tang HM, Mak KH, Hu S, Wang SS, Wong KM (2012). Cell survival, DNA damage, and oncogenic transformation after a transient and reversible apoptotic response. Mol Biol Cell.

[2] Kerr JF, Wyllie AH, Currie AR (1972). Apoptosis: a basic biological phenomenon with wide-ranging implications in tissue kinetics. Br J Cancer.

[3] Elmore S (2007). Apoptosis: a review of programmed cell death. Toxicol Pathol.

[4] Rehm M, Dussmann H, Janicke RU, Tavare JM, Kogel D, Prehn JHM (2002). Single-cell fluorescence resonance energy transfer analysis demonstrates that caspase activation during apoptosis is a rapid process. Role of caspase-3. J Biol Chem.

[5] Tyas L, Brophy VA, Pope A, Rivett AJ, Tavaré JM (2000). Rapid caspase-3 activation during apoptosis revealed using fluorescence-resonance energy transfer. EMBO Rep.

[6] Albeck JG, Burke JM, Aldridge BB, Zhang M, Lauffenburger DA, Sorger PK (2008). Quantitative analysis of pathways controlling extrinsic apoptosis in single cells. Mol Cell..

[7] Julien O, Wells JA (2017). Caspases and their substrates. Cell Death Differ.

[8] Reddien PW, Cameron S, Horvitz HR (2001). Phagocytosis promotes programmed cell death in C. elegans. Nature.

[9] Sun G, Guzman E, Balasanyan V, Conner CM, Wong K, Zhou HR (2017). A molecular signature for anastasis, recovery from the brink of apoptotic cell death. J Cell Biol.

[10] Tang HL, Tang HM, Fung MC, Hardwick JM (2015). In vivo CaspaseTracker biosensor system for detecting anastasis and non-apoptotic caspase activity. Sci Rep.

[11] Tang H, Tang H, Montell D (2013). Stress induced mutagenesis, genetic diversification, and cell survival via anastasis, the reversal of late stage apoptosis. Stress-induced mutagenesis.

[12] Ding AX, Sun G, Argaw YG, Wong JO, Easwaran S, Montell DJ (2016). CasExpress reveals widespread and diverse patterns of cell survival of caspase-3 activation during development in vivo. Elife.

[13] Kenis H, Zandbergen HR, Hofstra L, Petrov AD, Dumont EA, Blankenberg FD (2010). Annexin A5 uptake in ischemic myocardium: demonstration of reversible phosphatidylserine externalization and feasibility of radionuclide imaging. J Nucl Med.

[14] de Calignon A, Fox LM, Pitstick R, Carlson GA, Bacskai BJ, Spires-Jones TL (2010). Caspase activation precedes and leads to tangles. Nature.

[15] Levayer R, Dupont C, Moreno E (2016). Tissue crowding induces caspase-dependent competition for space. Curr Biol.

[16] Nakajima Y-I, Kuranaga E (2017). Caspase-dependent non-apoptotic processes in development. Cell Death Differ.

[17] Liu X, He Y, Li F, Huang Q, Kato TA, Hall RP (2015). Caspase-3 promotes genetic instability and carcinogenesis. Mol Cell.

[18] Cartwright IM, Liu X, Zhou M, Li F, Li C-Y (2017). Essential roles of Caspase-3 in facilitating Myc-induced genetic instability and carcinogenesis. Elife.

[19] Ichim G, Lopez J, Ahmed SU, Muthalagu N, Giampazolias E, Delgado ME (2015). Limited mitochondrial permeabilization causes DNA damage and genomic instability in the absence of cell death. Mol Cell.

[20] Florentin A, Arama E (2012). Caspase levels and execution efficiencies determine the apoptotic potential of the cell. J Cell Biol.

[21] Gong Y-N, Guy C, Olauson H, Becker JU, Yang M, Fitzgerald P, et al. ESCRT-III acts downstream of MLKL to regulate necroptotic cell death and its consequences. Cell. 2017;169:286–300.e16. doi:10.1016/j.cell.2017.03.020. 10.1016/j.cell.2017.03.020 PMC5443414 28388412

[22] Overholtzer M, Brugge JS (2008). The cell biology of cell-in-cell structures. Nat Rev Mol Cell Biol.

[23] Kaufmann SH, Earnshaw WC (2000). Induction of apoptosis by cancer chemotherapy. Exp Cell Res.

[24] Baskar R, Lee KA, Yeo R, Yeoh K-W (2012). Cancer and radiation therapy: current advances and future directions. Int J Med Sci.

